# A Plea for the Meatus Urinarius

**Published:** 1889-05

**Authors:** J. C. Olmsted

**Affiliations:** Atlanta, Ga.


VOL. VI. MAY, 1889. No. 3.

### Original Communications.

### A PLEA FOR THE "MEATUS URINARIUS."*

BY J. C. OLMSTED, M. D., ATLANTA, GA.

In this day of critical examination, scientific research and discovery, progressive thought marks the spirit of the age, and the medical profession, in its changing views and departures from old ideas and methods of practice, forms no exception to the rule, whatever cynics and pessimists may rail to the contrary.

Pre-eminently in the domain of surgery do we find bold innovations and radical changes (for the better in most respects), necessitated by a more enlightened pathology, with consequent means better adapted to accomplish the end. There follows naturally the upheaval of old plans and systems of treatment, rendered classic by time and the halo of illustrious names associated therewith. This is well, and cannot but redound to the advancement of our profession as a science, and the welfare of those en-

*Read before the Atlanta Society of Medicine.

trusted to our care; provided, that we exercise reasonable discrimination and judgment in our methods, and do not commit the error of assuming that the *newest* is always the *best.*

"Prove all things" is a very good maxim here, and it is desirable, while gladly welcoming that which promises good or better in the new, to also hold fast to that which has been tried and found good in the old. Bold, indeed, were the independent minds which first inaugurated many of these changes, venturing to dispute the conclusions of great men, whose names were as a shibboleth to the profession. Happily, the free spirit of enquiry which marks our times has made its influence felt even in our conservative profession; and the spectacle of an enlightened man of genius, attacked, ridiculed, and even persecuted, by jealous, book-bound worshippers of great names, (or their own small ones,) is seldom witnessed; and when seen, is marked only to be condemned.

The pendulum now swings to the opposite extreme, indeed, and we now "seek only to do, hear and tell some new thing." Pioneers in every department abound, from the adventurous soul who proposes to "resect" a degenerate lung, to him who would rid the human organism of the "baccilus tuberculosis" with bi-chloride of mercury! Too many, unfortunately, are found who are prone to rush headlong into everything new that is proposed, provided that it has the sanction of some great operator's name, and that brilliant results in his hands have been achieved. The long experience, matured judgment and manual dexterity of the originator and his equals, are too often ignored in the calculation, and that "we are all equals" is assumed.

"What crimes have been committed in thy name," might well be said in too many cases of this kind. The tendency now is to medical "fashions" in treatment, and frequently a good operation is brought into disrepute by its indiscriminate application to all cases that may possibly suggest it; one idea appearing to take possession, to the exclusion of all other considerations, and a just appreciation in all of their bearings of the conditions to be treated. Especially has this appeared to be true, in my observation, concerning the subject which I have selected for a few remarks this evening.

When the influence of a contracted or strictured meatus, as a cause of spasmodic stricture, simulating organic stricture in the deep urethra, was first demonstrated prominently by Dr. Otis; when, by the same authority and by others, the effects of the above mentioned condition, as exhibited in various reflex disturbances of the genito-urinary system, or in certain neuroses of the organism, more or less remote, were made apparent, a flood of light was thrown upon subjects which had previously been most obscure, baffling the skill of the able surgeon and practitioner, and contributing in no small degree to the opprobrium of surgery and medicine.

An operation so simple as that indicated for the relief of these conditions, and in urethral and bladder troubles, often dispensing with bloody and dangerous operations in the deeper urethra, (as were often done for strictures which never existed, save as reflex spasmodic contraction,) could not but fascinate those who witnessed these remarkable results, and create with many, in their enthusiasm, a tendency on the presentment of a case exhibiting symptoms of genito-urinary disturbance, or nervous disorders of somewhat obscure character, and pointing to a possible reflex origin of the kind mentioned, to at once ascribe all such difficulties to an offending meatus urinarius. If upon examination, the meatus is found (as it usually is) somewhat smaller than the canal behind it, this settles the matter, without further enquiry often, and an immediate incision is made.

"Slitting of the meatus," for nearly anything and everything, has become so common that it is the glib phrase upon the tongue of nearly every first-course student, whose fingers itch to grasp the bistoury. I would not be understood as underrating this valuable operation, when I say in my opinion it is too frequently performed when not indicated. Nor is this all; but the improper manner in which it is often performed when indicated, is not, in my judgment, a matter of indifference; entailing, as it frequently does, great annoyance and discomfort to the patient for the balance of his life, and failing to relieve symptoms, for reasons to be presently mentioned.

As I shall have occasion to animadvert further upon this phase of my subject, I desire not to be misunderstood. I think I have a full and proper appreciation of this operation, when properly performed, upon the class of cases requiring it. In common with many of my brethren, I have obtained the most gratifying results, and have seen difficulties and apprehended dangers cleared away by this simple procedure in the most surprising and satisfactory manner. So much for my estimate of "meatotomy."

To return to the abuses of it, upon which I was animadverting, I apprehend from the number of cases which have come under my observation, with wide, gaping, irritated urethral orifices, resembling often an hypospadius, that there are some amongst us "who have a zeal not according to knowledge." The unfortunate objects of this zeal usually come seeking relief from the sufferings for which this operation was originally performed; and complain that, in addition to former woes, they are annoyed with "sprazzling urine," soreness at the orifice, etc. Examination in such cases revealed that the hypospadic slit that had been made had not reached the real source of the original troubles, namely, a tough little fibrous ring or stricture, from one-fourth to one-half an inch, sometimes an inch, behind the meatus. This it was that required free and deep incision; the meatus itself calling for but comparatively slight incision. The indomitable "slitter" had cut down at times *to* but not *through* this, from the inferior commissure of the meatus, and had not gone farther, for the reason, I suppose, that even he had to stop somewhere, and the meatus was gaping inquiringly at him, and ready, apparently, to swallow a "polished gas-pipe."

This unnatural and widely gaping orifice may seem a trivial matter to the operator, but it is not usually so regarded by the owner of it; who, as I have said, complains that he is unable to project his stream satisfactorily and naturally; that his urine "sprazzles," frequently wetting his person, etc. Meati of this character often present a red, irritated-looking mucous membrane, reminding one of an hypospadius (which, indeed, it practically is). The altered shape of the meatus, together with the loss of the natural muscular power of contracting properly, being responsible for the conditions complained of. The urethra, it should not be forgotten, is not a merely passive tube, transmitting urine from the bladder, and semen from the vesiculæ seminales and prostate gland, by the *vis a tergo*, supplied by the peculiar muscular mechanisms characteristic of those structures. These, while the chief agents in those physiological processes, are not alone concerned. The urethra itself, by the arrangement of the plain muscular fibres which surround it, throughout its course possesses an independent power of contracting upon its contents. Again, the shape and size of the meatus must, upon mechanical principles, have a profound effect upon the direction and force with which the urine, particularly, is expelled.

I am not fond of using mechanical figures with which to illustrate physiological processes, but in this connection would recall the effect of the hose narrowing towards its nozzle upon the delivery of its jet of water. In somewhat like manner, and within anatomical and physiological limits, does the urethra, narrowing at the meatus, exercise its function. And here, with all diffidence and becoming respect for that eminent authority, I must take issue with the teachings of Prof. F. N. Otis.

That original investigator and accomplished surgeon denies that the urethra narrows at the external orifice, and he asserts that the normal or typical meatus urinarius is of the "same calibre as the canal behind it." He also, in accordance with this view, holds that that portion of the urethra situated within the glans, called and described by anatomists the "fossa navicularis," does not exist normally; it is abnormal, in his opinion. He bases his views, apparently, upon the following facts, which he has observed:

First, he claims to have found in two or three cases (out of one hundred, in which exemption from previous inflammatory disorders was asserted,) a meatus urinarius which corresponded in its calibre with that of the "canal behind it." This, therefore, is the "normal" or "typical" meatus.

Now, while I am familiar with the variations and deviations from any fixed standard, at this interesting anatomical point it seems to me that if in one hundred urethras, claimed to be free from previous disease, Dr. Otis only finds "two or three," (I quote his figures and his own words,) that he takes the "exception for the rule," unless we are prepared to admit that nature does not usually know what she is about, and only succeeds two or three times out of a hundred in forming this part correctly.

Dr. Otis appears to hold that with the "fall of Adam" the meatus fell from the 'highest normal type" of an "even calibre with that of the canal behind it," to its present degenerate and narrower state of existence; and its transgression is thus the origin of the fossa naviculasis.

He says that he does not doubt that if our "Great Progenitor" could have been examined with a bulb sound, "that his meatus would have been found to correspond with the size of the canal behind it; but that now, after six or more thousand years, indiscretions and irregularities having crept into the world, the meatus urinarius, amongst other things, has varied from its normal type," etc.

In this account of the "evolution of the meatus," as we see it, it seems that, for once, at any rate, religion and science are harmonized! It follows from the foregoing, therefore, that Dr. Otis regards the narrowing of the urethral canal at the meatus, which is usually found, as abnormal; a "stricture" at that point; and this in cases claimed to be free from antecedent inflammatory action there. The reason why such strictured meati usually give no trouble is, he says (and I agree with him in the statement,) "that when the muscular structure of the meatus and the urethra behind it is in perfect condition, it is enabled to empty the urethra completely after urination; but let inflammatory action be set up in this locality, as may occur from extension of an infantile or an adult balaritis, or from gonorrhœa, or from any other cause, and a plastic exudation results, which, becoming organized, disables the urethral muscular structure at this point; it is no longer able to act efficiently in expelling the last drops of urine; they are retained, dribbling results," etc., etc.

In all of this statement concerning the *pathology* of a *diseased*

or *strictured* meatus, I fully concur, but it does not, in my judgment, prove that those ninety-seven or ninety-eight cases out of the hundred, free from such inflammatory antecedents, and yet having meati narrower than "the canal behind them" are abnormal, but the contrary. I think that the facts go to prove that there is a normal narrowing at this point anatomically, and I believe that it is intended so to be by nature, for the effect it has upon the clear projection of the urine in its exit from the body, of which I earlier made mention.

In regard to the fossa navicularis, or that dilated portion of the urethral canal behind the meatus, the existence of which "normally" Dr. Otis also denies, I think it is the result of the functional exercise of the urinary canal; the urethra being dilated at this point to some extent by the natural pressure of the stream as it encounters some *natural* resistance at the narrowing meatus, intended for the direction (and to some extent for the force) of the stream in its expulsion. When the meatus and the muscular structures at this point and its immediate vicinity, have been impaired by inflammatory action, (as so admirably described by Dr. Otis,) there results an unnatural obstacle and friction to the stream, which causes an unnatural bulging and exaggerated enlargement of this fossa, disturbing its natural proportions.

Another fact claimed by Dr. Otis is that examinations of the fœtus and newly-born prove that this fossa does not exist, "but is an acquired condition." This does not invalidate my argument that it is also a natural condition, being, as previously stated, the result of the functional exercise of the urinary canal. Appeal made to fœtal structures, in support of theories contradictory of adult conditions in dispute, is not always a safe method of reasoning. Indeed, some very startling claims might be postulated upon this method. But I accept the statement of Dr. Otis, that the fossa naviculasis is not found in the fœtus or newly-born. This statement, as made in his work on "Stricture of the Male Urethra," appears to be based upon observations made by Prof. Thos. R. Brown, of Baltimore. Professor Brown was investigating this point, as to the existence of the fossa navicularis. He says that he "examined a number, and in not one did this dilatation occur." He further remarks, (and I accept this statement, also,) "in all of these examinations, the meatus was *invariably* found to be *narrower* than the rest of the canal." In regard to the latter fact it appears to me that the appeal made by Dr. Otis to fœtal structures, in regard to the fossa navicularis, certainly proves that the meatus is narrower than the canal behind it. Once granted that the meatus is narrower than the canal behind it, it is easy to understand how the fossa navicularis comes into existence by functional use of the canal, as previously described. The fact that this meatus, normally narrower than the "canal behind it," may become abnormally contracted by inflammatory action, and thus create trouble; or that, in some instances, it may be congenitally too small, or badly shaped, and give rise to an abnormal bulging of the fossa naviculasis, is no proof that normally the meatus is not narrower in its calibre than "the canal behind it," or that there is not normally a fossa navicularis.

All are familiar with the troubles that may arise from abnormal conditions of the prepuce, yet most of us, I opine, will admit that the prepuce, as such, is a normal structure.

In conclusion, as a matter of practical fact, most of the urethræ which are presented to us are diseased, and apply for treatment. The meati which we encounter have been injured and contracted by inflammation, as a general rule. In the majority of cases the meatus requires incision more or less, according to the amount of contraction existing. But the widely gaping orifices so frequently encountered are not necessary, are not imitations of nature, and hence are eminently unsurgical. I protest against such perversions and abominable botches being represented as "performed after the method of Otis."

To hold that eminent and successful surgeon as responsible for such work as this, would indeed be as unjust as it is untrue. I have ventured to criticize the views of Dr. Otis upon certain anatomical points, as being extreme, and, in my humble judgment, not warranted by facts or proven by his arguments. I have done so because legitimate criticism is in order here, and I think it unwise and destructive of true progress for our admiration and appreciation of genius to lead us to "call a man master," to the exclusion of independent thinking and criticism. I cannot refrain from adding that, in my opinion, and that of many others of more weight and value, that this remarkably gifted and original man has done more for urethral surgery (and incidentally for lithotrity,) than any man of this age; and to those who are familiar with him as an operator in his specialty, his wonderful delicacy of touch, and marvelous dexterity in the manipulation of instruments, has often suggested the workings of some great magician.

